# Supplementary material for: Evaluation of participant reluctance, confidence, and self-reported behaviors since being trained in a pharmacy Mental Health First Aid initiative
Source: PLoS One. 2020 May 4;15(5):e0232627. doi: 10.1371/journal.pone.0232627 (PMC7197798; doi:10.1371/journal.pone.0232627)
Supplement: S1 File — (DOCX) [file pone.0232627.s001.docx]

2019 Mental Health First Aid Evaluation Survey

Start of Block: Default Question Block

Q1 We invite you to participate in a research study being conducted by investigators from The University of Iowa. The following is a brief survey about your experiences and thoughts regarding the Mental Health First Aid (MHFA) training you completed in 2018. The purpose of the survey is 1) to understand how you use MHFA techniques in practice, 2) to collect feedback about the MHFA training in order to further improve it, and 3) to gather information that will help us to address your needs and concerns regarding the use of MHFA.  We will use your responses to understand how we can best support pharmacy professionals like you who have been trained in MHFA. Your responses also will help pharmacy stakeholders and others better understand the potential impact of training pharmacy professionals in MHFA   If you agree to participate, we would like you to complete the following anonymous survey about MHFA, which will take approximately 10 minutes.  It will not be possible to link you to your responses on the survey. Taking part in this research study is completely voluntary.  If you do not wish to participate in this study, please close your web browser window now or at any time before submitting the survey.

If you have questions about this survey, or do not want to be contacted further please email:

Matthew Witry matthew-witry@uiowa.edu

Anthony Pudlo apudlo@iarx.org

If you have questions about the rights of research subjects, please contact the Human Subjects Office, 105 Hardin Library for the Health Sciences, 600 Newton Rd, The University of Iowa, Iowa City, IA  52242-1098, (319) 335-6564, or e-mail irb@uiowa.edu.   This survey includes questions related to suicide prevention. The national suicide prevention lifeline number is 1-800-273-8255.   Thank you for your feedback.

End of Block: Default Question Block

Start of Block: Block 1

Q2 When did you participate in MHFA training?

- January – March 2018
- April – June 2018
- July – September 2018
- October – December 2018
- Other (Please specify) ________________________________________________

Q3 Please rate your level of agreement or disagreement with the following statements related to your beliefs about mental health crisis and suicide.

|  | Strongly disagree | Somewhat disagree | Neither agree nor disagree | Somewhat agree | Strongly agree | Not applicable |
| --- | --- | --- | --- | --- | --- | --- |
| There is very little that I can do to help if someone thinking about suicide doesn’t acknowledge the situation. |  |  |  |  |  |  |
| If someone contemplating suicide does not seek assistance, there is nothing I can do to help. |  |  |  |  |  |  |
| If someone in a mental health crisis refuses to seek help, it should not be forced upon then. |  |  |  |  |  |  |
| I cannot understand why anyone would contemplate suicide. |  |  |  |  |  |  |
| I am too busy to provide mental health first aid at work. |  |  |  |  |  |  |
| I do not know most patients well enough to know when they are in a mental health crisis. |  |  |  |  |  |  |

Q4 Please rate your level of agreement or disagreement with the following statements related to your confidence level in your ability to perform mental health first aid (MHFA) including the following skills now.   I am confident I can....

|  | Strongly disagree | Somewhat disagree | Neither agree nor disagree | Somewhat agree | Strongly agree | Not applicable |
| --- | --- | --- | --- | --- | --- | --- |
| Recognize the signs that someone may need MHFA. |  |  |  |  |  |  |
| Ask someone if they are thinking about suicide. |  |  |  |  |  |  |
| Listen non-judgmentally to someone experiencing a mental health crisis. |  |  |  |  |  |  |
| Offer basic "first aid" level information to someone experiencing a mental health crisis. |  |  |  |  |  |  |
| Offer reassurance to someone experiencing a mental health crisis. |  |  |  |  |  |  |
| Encourage someone experiencing a mental health crisis to seek professional help. |  |  |  |  |  |  |
| Encourage self-help strategies for someone experiencing a mental health crisis. |  |  |  |  |  |  |

Q5 Please report your best estimate of the number of times that you have had experience with the situation since completing MHFA training.

|  | 0 | 1 | 2 | 3 | 4+ | Not applicable |
| --- | --- | --- | --- | --- | --- | --- |
| Thought someone’s behavior might indicate they are having a mental health crisis. |  |  |  |  |  |  |
| Thought someone’s behavior might indicate they are having suicidal thoughts. |  |  |  |  |  |  |
| Asked someone about their distressed mood. |  |  |  |  |  |  |
| Asked someone if they are considering suicide. |  |  |  |  |  |  |
| Listened non-judgmentally to someone experiencing a mental health crisis. |  |  |  |  |  |  |
| Referred someone to appropriate resources because you were concerned they might be experiencing a mental health crisis. |  |  |  |  |  |  |
| Referred someone to appropriate resources because you were concerned they were considering suicide. |  |  |  |  |  |  |
| Engaged with a mental health crisis resource on behalf of someone. |  |  |  |  |  |  |
| Engaged with emergency medical or police services because of someone experiencing a mental health crisis. |  |  |  |  |  |  |

Q6 Without using any identifying information, do you have any experience with MHFA you wish to share?

________________________________________________________________

________________________________________________________________

________________________________________________________________

________________________________________________________________

________________________________________________________________

Q7 What major challenges or problems have you faced while using MHFA in your role as a pharmacist?

________________________________________________________________

________________________________________________________________

________________________________________________________________

________________________________________________________________

________________________________________________________________

Q8 What improvements would you recommend for future MHFA training recruitment?

________________________________________________________________

________________________________________________________________

________________________________________________________________

________________________________________________________________

________________________________________________________________

Q9 What suggestions would you propose related to how MHFA training is delivered?

________________________________________________________________

________________________________________________________________

________________________________________________________________

________________________________________________________________

________________________________________________________________

Q10 What suggestions would you propose for further supporting pharmacists in the area of mental healthcare? What do you still need help with?

________________________________________________________________

________________________________________________________________

________________________________________________________________

________________________________________________________________

________________________________________________________________

Q11 Is there anything else on this topic not included in the previous questions that you feel important to tell us?

________________________________________________________________

________________________________________________________________

________________________________________________________________

________________________________________________________________

________________________________________________________________

Q12 What is your gender identity?

- Male
- Female
- Please specify ________________________________________________
- Prefer not to answer

Q13 How old are you?

- Below 25
- 25-34
- 35-44
- 45-54
- 55-64
- Above 65

Q14 How would you describe your role?

- Practicing pharmacist
- Student pharmacist
- Pharmacy technician
- Pharmacy school faculty
- Other (Please specify) ________________________________________________

Q14a What year in pharmacy school were you when you completed MHFA training?

- P1
- P2
- P3
- P4

Q15 How would you describe your work setting?

- Community – independent
- Community – chain
- Community – grocery
- Hospital/Health System
- College or University
- None
- Other (Please specify) ________________________________________________

Q15a How many years of work experience do you have as a pharmacist?

________________________________________________________________

Q15b How many years of work experience do you have as a pharmacy technician?

________________________________________________________________

Q15c How many years of work experience do you have as a faculty member?

________________________________________________________________

Q15d How many years of work experience do you have as a pharmacy technician or intern, including before you started pharmacy school?

________________________________________________________________

Q15e How many years of work experience do you have in your current role?

________________________________________________________________
