# Supplementary material for: Evaluation of participant reluctance, confidence, and self-reported behaviors since being trained in a pharmacy Mental Health First Aid initiative
Source: PLoS One. 2020 May 4;15(5):e0232627. doi: 10.1371/journal.pone.0232627 (PMC7197798; doi:10.1371/journal.pone.0232627)
Supplement: Attachment — Submitted filename: PLoS RR 4_14_2020.docx [file pone.0232627.s003.docx]

PONE-D-19-33742R1
Evaluation of participant reluctance, confidence, and self-reported behaviors since being trained in a pharmacy Mental Health First Aid initiative
PLOS ONE

Thank you for the opportunity to revise this manuscript.

Please consider the comments from reviewer 2 and acknowledge any limitations not already included and resubmit.

**Comments to the Author**

Reviewer #1: (No Response)

Reviewer #2: All comments have been addressed

Reviewer #5: All comments have been addressed

2. Is the manuscript technically sound, and do the data support the conclusions?

Reviewer #1: Partly

Reviewer #2: Yes

Reviewer #5: Yes

 3. Has the statistical analysis been performed appropriately and rigorously?

Reviewer #1: Yes

Reviewer #2: Yes

Reviewer #5: Yes

4. Have the authors made all data underlying the findings in their manuscript fully available?

Reviewer #1: Yes

Reviewer #2: Yes

Reviewer #5: (No Response)

 5. Is the manuscript presented in an intelligible fashion and written in standard English?

Reviewer #1: Yes

Reviewer #2: Yes

Reviewer #5: Yes

 6. Review Comments to the Author

Please use the space provided to explain your answers to the questions above. You may also include additional comments for the author, including concerns about dual publication, research ethics, or publication ethics. (Please upload your review as an attachment if it exceeds 20,000 characters)

Reviewer #1: Thank you for your work to address the comments made by your reviewers. Your time, consideration of these requests and efforts are greatly appreciated. However, based on my concerns and concerns that were raised by at least two other reviewers regarding the validity of your survey tool/findings, I cannot recommend publication. While MHFA and other mental health roles are growing in community pharmacy practice and it is important to understand the impact of these trainings, I feel the analysis is too focused on self-reported post training and did not include a baseline assessment that hinders its findings.

*This is a limitation which we discuss.*

My greatest concern was that a validated survey tool was not used -- there are several validated attitude scales regarding suicide (ATTS, Understanding of Suicidal Patients Scale) that could have been used. Further, a qualitative framework could have been used (such as the Theory of Planned Behavior) if you felt that the validated surveys were not a good fit.

*We feel we used reasonable constructs with reluctance and confidence. A knowledge inventory like ATTS would be even less useful without a pre-test. With reluctance and confidence one could look at the responses and make a reasoned interpretation that someone reporting these attitudes are confident and are not reluctant. Which we believed were the best constructs given we were unable to do a pre-test.*

Additional comments:
1) LIne 71 -- suggest using language suggested by APhA community-based

*Adopted.*

2) Suggest including reference to the survey in your methods if intended to be included in your published article

*I’m not sure what this refers to, we do reference the articles that we used for our adapted instrument.*

3) Domains unclear (Line 120). Only mention demographics and then . --> suggest listing all domains and then moving into description of each

*This has been revised, thank you, this makes it read more clearly.*

4) Time since training being 6-18 months. I feel the survey not being consistently conducted xx amount of time since being completed is a significant limitation as this could impact retention of concepts, how much they are using, etc

*We did already have this as a limitation, but we have written additional text to make this limitation more explicit.*

5) Relevance of data in lines 234-239 since the national behavorial council created the training and is concerned with fidelity and does not allow adjustments to training?

*We believe these are compatible with the training guidelines. Such as, include more people like technicians and have these trainings at work.*

Reviewer #2: All comments have been appropriately addressed, appreciate author responses to reviewer recommendations.

Reviewer #5: I think the authors addressed my primary concerns. I will say there may be still some typos like line 130 "participants". I am still not entirely convinced how knowledge is reluctance but will accept their attempt here. I might have suggested that they just refer to reluctance and not view it as

*Yes, we have removed all references to the reluctance items being knowledge.*
